# Inhaled Corticosteroids Prescribed for COPD Patients with Mild or Moderate Airflow Limitation: Who Warrants a Trial of Withdrawal?

**DOI:** 10.2147/COPD.S238239

**Published:** 2019-12-31

**Authors:** Timothy H Harries, Gill Gilworth, Christopher J Corrigan, Patrick B Murphy, Nicholas Hart, Mike Thomas, Patrick T White

**Affiliations:** 1 Department of Public Health and Primary Care, School of Population Health and Environmental Sciences, King’s College London, London, UK; 2 Department of Asthma Allergy and Respiratory Science, King’s College London, London, UK; 3 Lane Fox Respiratory Unit, Guy’s and St. Thomas’ NHS Foundation Trust and King’s College London, London, UK; 4 Primary Care and Population Medicine, University of Southampton, Southampton, UK

**Keywords:** pulmonary disease, chronic obstructive, inhaled corticosteroids, drug withdrawal, mild airflow limitation, moderate airflow limitation

## Abstract

COPD patients prescribed inhaled corticosteroids (ICS) outside guidelines should be targeted for ICS withdrawal. Within a primary care population of 209,618 we used a combination of digital search algorithm, individual record review, and clinical review to identify COPD patients suitable for a trial of ICS withdrawal. At most, 39% of COPD patients with mild or moderate airflow limitation prescribed ICS were suitable for withdrawal according to Global Initiative for Chronic Obstructive Lung Disease (GOLD) guidelines. Recurrent exacerbations and reversible airway obstruction were the main reasons for patients’ unsuitability for withdrawal. Identifying COPD patients in whom ICS withdrawal should be considered presents a challenge to primary care clinicians.

## Introduction

Many patients with chronic obstructive pulmonary disease (COPD), without asthma, are treated with inhaled corticosteroids (ICS) without reference to guidelines.^1^,^2^ ICS usage increases the risk of complications including pneumonia.^3^ Most patients who receive inappropriate ICS are prescribed them within primary care. These patients should be targeted with a view to ICS withdrawal. In primary care in England, 24% of COPD patients were prescribed ICS and long-acting beta-agonists (LABA) outside of the 2011 Global Initiative for Chronic Obstructive Lung Disease (GOLD) guidelines.^1^ Between 2007 and 2010, large increases in ICS prescribing were not associated with expected impact on the incidence of admissions for exacerbations.^4^,^5^ We have found that patients prescribed ICS outside guidelines are happy to consider their withdrawal.^6^

The most recent GOLD guidelines suggest criteria for commencing ICS and LABA as initial treatment in patients who exhibit Grade D COPD and are symptomatic with a blood eosinophil count ≥300 cells/µL.^3^ GOLD offers the option of the addition of ICS to patients already using LABA who continue to have exacerbations at any frequency with a blood eosinophil count ≥300 cells/µL, or who experience ≥2 moderate exacerbations per year or at least one severe exacerbation in the prior year with a blood eosinophil count ≥100 cells/µL. GOLD recommends considering ICS withdrawal from those who develop pneumonia or show “lack of response,” the latter undefined.^3^ Meta-analyses report that ICS withdrawal is not associated with increased risk of COPD exacerbations, despite heterogeneity in exacerbation definition and withdrawal criteria.^7^,^8^

Clinical algorithms for ICS withdrawal include that of Miravitlles et al who categorised COPD patients into three groups according to their predicted exacerbation risk following withdrawal.^9^ By their analysis those without asthma, with an FEV_1_ >50% predicted and no prior exacerbations should have ICS withdrawn. Those with features suggestive of concurrent asthma and a history of exacerbations in the previous year should continue ICS use. A third group, thought not to have asthma, either with an FEV_1_ >50% predicted and a history of exacerbations, or with an FEV_1_ <50% predicted and no prior exacerbations, may warrant ICS withdrawal but require close follow up.

### Aim

To identify COPD patients with mild or moderate airflow limitation (FEV1 ≥50% predicted), no post-bronchodilator reversibility (<15%), and prescribed ICS outside the 2019 GOLD guidelines, who warrant a trial of ICS withdrawal.

## Materials and Methods

We devised a digital search algorithm and conducted a survey of electronic patient records within primary care to identify COPD patients with mild or moderate airflow limitation, without asthma, who had been prescribed ICS at a dose greater than beclomethasone 400 µg/day or an equipotent dose of ICS in the prior 4 months. The algorithm identified patients with a diagnosis of COPD, no previous diagnosis of asthma, and an FEV_1_ at least 45% of the predicted value measured in the previous year. We field tested the search algorithm in two general practices. We rolled out the search across practices within two London Clinical Commissioning Groups (CCGs). We searched, individually, the medical records of patients identified by the algorithm, examining prescribing history, investigations including spirometry results, correspondence with secondary care, and free-text recording of consultations. Eligible patients were invited by their general practitioner (GP) to attend a COPD review to consider ICS withdrawal. At review by a research GP, spirometry with assessment of reversibility was undertaken and exacerbation history assessed. Patients were excluded if ICS prescription was in accordance with GOLD guidelines.^3^

## Results

The records of 20 London general practices with a total patient population of 209,618 were searched using the digital algorithm, of which 2967 patients had a recorded COPD diagnosis (1.42% prevalence). Of these, 392 patients were identified as potential eligible candidates for ICS withdrawal. Upon individual record review, 65 patients had evidence of severe airflow limitation (FEV1<50% predicted) not detected by the algorithm. Of the remaining 327 patients with mild or moderate airflow limitation, 86 (26%) had a record of disease exacerbations (≥2 moderate or 1 severe) in the prior year. Fifteen patients (5%) had a record of reversibility of airway obstruction (FEV_1_ reversibility ≥15%). In 77 patients (24%) there were additional issues making them unsuitable for withdrawal in primary care including lung cancer, dementia, housebound, and some who had undergone recent ICS withdrawal. Inconsistencies in diagnosis and exacerbation recording were frequently seen in patient notes. Repeat prescriptions of antibiotics and prednisolone (rescue packs) were often provided without corroborating evidence of an exacerbation.

Of the patients identified as potentially suitable for ICS withdrawal, 149 were invited for review. Sixty-one (19% of the 327 potential candidates with mild or moderate airflow limitation after individual record review) attended. At review 10 patients (3%) had reversible airway obstruction, while 2 patients (<1%) had a history of either 1 severe or 2 moderate exacerbations within the past year. Nine patients (3%) had either severe airflow limitation or normal spirometry and thus were ineligible for this study. Forty patients provided consent and proceeded to a trial of ICS withdrawal.

Eighty-eight (27%) patients did not respond to invitation for assessment. In the unlikely event that all 88 non-responders had been unsuitable for a trial of ICS withdrawal, then 40 patients (12% of the 327 potential candidates) would have been suitable. Conversely, if all 88 non-responders had been suitable, 128 (39%) of the 327 patients identified from individual record and clinical review would have warranted a trial of ICS withdrawal according to GOLD guidelines.

## Discussion

A small proportion of COPD patients with mild or moderate airflow limitation prescribed ICS may be suitable for withdrawal if current GOLD guidelines are applied.^3^ The recording of COPD exacerbations and airflow reversibility, key determinants of suitability for ICS prescription, are inconsistent or absent in primary care clinical records. At most 39% of COPD patients prescribed ICS outside guidelines would warrant a trial of withdrawal when identified by a combination of a digital search algorithm, individual record review, and clinical review.^3^ The clinical benefit to patients, the potential negative effect on airflow limitation, and the cost to health services of intensive efforts to withdraw ICS from patients with mild or moderate airflow limitation need to be evaluated. The decision to withdraw ICS should be dependent on the history of exacerbations, based on the Anthonisen criteria as recommended by the GOLD guidelines.^3^,^10^ This history should be sought at the COPD review in primary care. Inconsistencies in the recording of exacerbations in the clinical record may prevent confirmation of a patient’s reported exacerbation history and undermine the accuracy of this assessment. Identification and definition of patients in whom ICS withdrawal should be considered present a difficult and potentially costly challenge to primary care clinicians who have responsibility for continued ICS prescribing.

## Data Sharing Statement

The data upon which this analysis was based are available in anonymised form, upon receipt of an appropriate request.
